# Bioactivity, Compounds Isolated, Chemical Qualitative, and Quantitative Analysis of *Cymbaria daurica* Extracts

**DOI:** 10.3389/fphar.2020.00048

**Published:** 2020-02-07

**Authors:** Xue Gong, Jie Wang, Meiying Zhang, Peng Wang, Congcong Wang, Ruyu Shi, Erhuan Zang, Mingxu Zhang, Chunhong Zhang, Minhui Li

**Affiliations:** ^1^ Department of Pharmacy, Baotou Medical College, Baotou, China; ^2^ Clinical Laboratory, The First Affiliated Hospital of Baotou Medical College of Inner Mongolia University of Science and Technology, Baotou, China; ^3^ Pharmaceutical Laboratory, Inner Mongolia Autonomous Region Academy of Chinese Medicine, Hohhot, China; ^4^ Guangxi Key Laboratory of Medicinal Resources Protection and Genetic Improvement, Guangxi Botanical Garden of Medicinal Plants, Nanning, China; ^5^ Inner Mongolia Key Laboratory of Characteristic Geoherbs Resources Protection and Utilization, Baotou Medical College, Baotou, China

**Keywords:** *Cymbaria daurica* L., Scrophulariaceae, anti-inflammatory activity, inhibition of α-glucosidase activity, UHPLC-Q-Exactive Orbitrap HRMS, phenylethanoid glycoside, verbascoside

## Abstract

*Cymbaria daurica* L. is widely used in traditional Mongolian medicine for the treatment of impetigo, psoriasis, pruritus, fetotoxicity, and diabetes. Therefore, the anti-inflammatory and α-glucosidase-inhibitory activities of four polar *C. daurica* extracts (water, n-butanol, ethyl acetate, and petroleum ether extract) were preliminarily evaluated to identify the active extracts. We also investigated the chemical composition of the active extracts by phytochemical analysis. The n-butanol and ethyl acetate extracts exhibited significant (*p* < 0.05) anti-inflammatory activities by inhibiting lipopolysaccharide-induced nitric oxide (NO) production in RAW 264.7 cells. None of the tested extracts exhibited cytotoxic effects at the effective concentrations. The ethyl acetate extract significantly inhibited α-glucosidase activity, and the inhibition potency was equivalent to that of acarbose (*p* > 0.05). The n-Butanol extract presented the second highest inhibitory activity. As the n-butanol and ethyl acetate extracts were found to have potent anti-inflammatory and α-glucosidase-inhibitory activities, we separated and identified 10 compounds from the extracts. Among them, vanillic acid, cistanoside F, echinacoside, arenarioside, verbascoside, isoacteoside, and tricin were isolated from *C. daurica* for the first time. Further, 30 compounds from the n-butanol and ethyl acetate extracts of *C. daurica* were identified using UHPLC-Q-Exactive. The present study demonstrates for the first time that *C. daurica* contains phenylethanoid glycosides. In addition, this novel HPLC method was subsequently used for simultaneous identification of five compounds in the n-butanol and ethyl acetate extracts of *C. daurica*. This study provides a chemical basis for further characterization and utilization of *C. daurica,* which could be a potential source of novel anti-diabetic and anti-inflammatory agents.

## Introduction

Mongolian medicines, which are being used for decades to manage various diseases (Zhang et al., 2015), have a history of more than 1,000 years, and Mongolians have developed their system of medicines based on their own culture and experience (Li et al., 2012). Treatment of diseases with Mongolian medicine-derived compounds seems to be highly attractive because of their accessibility and ease of isolation (Zhang et al., 2015). Thus, Mongolian medicine is considered important in drug discovery. Furthermore, it has gradually gained interest as a valuable source of potential medicines.

**Figure 1:**
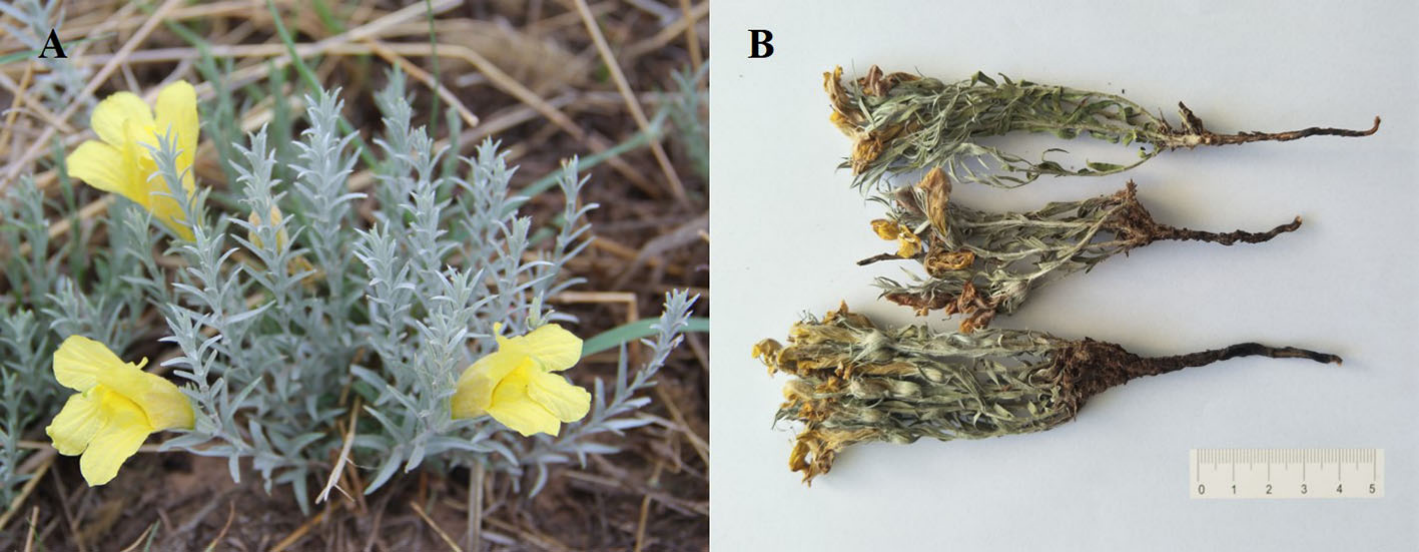
Images of *Cymbaria daurica* from aerial parts of *C. daurica* **(A)** and the whole plant of *C. daurica* **(B)**.

*Cymbaria daurica* L. (**Figure 1**) belongs to the family Scrophulariaceae and is an important component of Mongolian medicines (Zhang et al., 2013). According to the *Mengyaozhengdian*, a complete and systematic Mongolian pharmaceutical classic of the 19th century, the whole plant is widely used in traditional Mongolian medicines for impetigo, psoriasis, pruritus, fetotoxicity, and diabetes (Health Department of Inner Mongolia Autonomous Region, 1987; Pharmacopoeia Committee of the Ministry of Health of the People’s Republic of China, 1998; Zhang et al., 2013; Chang et al., 2015). In addition, *C. daurica* has anti-inflammatory, anti-bacterial, and anti-oxidant activities (Zhang et al., 2013; Hu, 2018). *C. daurica* has been extensively used in several classic herbal formulations recommended for anti-inflammation, such as *Siweixinbasan* and *Baweixinbasan* (Zhang et al., 2013; Liang et al., 2016). Some researchers have explored the pharmacological activities of *C. daurica*. The anti-inflammatory activities of *C. daurica* ethanol extracts have been reported in xylene-induced ear edema KM mice and egg white-induced paw edema SD rats. These studies revealed that *C. daurica* ethanol extract could restrain ear edema in mice and paw edema in rats at 6 h after egg white-induced inflammation (Guo et al., 2017). Chang et al. (2015) showed that the water and n-butanol extracts of *C. daurica* significantly reduced glucose levels in alloxan-induced diabetic mice. However, there are only a few phytopharmacological studies on *C. daurica*. The main pharmacological activities of *C. daurica* can be attributed to the different bioactive compounds previously reported in this traditional plant (Amessis-Ouchemoukh et al., 2014). However, the chemical composition of *C. daurica* is not entirely clear (Li et al., 2014).

Therefore, to validate the therapeutic effects of *C. daurica*, we investigated the anti-inflammatory and α-glucosidase-inhibitory activities of various extracts (water extract, n-butanol extract, ethyl acetate extract, and petroleum ether extract) of *C. daurica* to identify the active extracts. Next, the compounds were separated from the active extracts. Combined with the results of previous phytochemical research (Li et al., 2014; Bai et al., 2018), a database of *C. daurica* compounds was constructed; the chemical composition of the extracts was analyzed by UHPLC-Q-Exactive. In addition, the components with a high content and potential activity in *C. daurica* were simultaneously determined using a novel high-performance liquid chromatography (HPLC) method.

## Materials and Methods

### Materials

#### Plant Material

*C. daurica* was collected in grassland (44°34′5.8″ N, 117°35′6.7″ E, Elevation: 1058.9 m), Haoletugaole Town, West Ujimqin Banner, XilinGol League, Inner Mongolia Autonomous Region in June 2018 and was authenticated by Life Science Faculty, Inner Mongolia University. Furthermore, voucher specimen of the same was deposited in the Herbarium of HIMC (Specimen No.: 152500180629025LY) for future reference.

#### Cells and Reagents

Raw 264.7 cell line was purchased from the Cell Bank in the China Academy of Science (Shanghai, China). High glucose Dulbecco’s modified Eagle medium (DMEM) (Gibco, USA), fetal bovine serum (FBS), penicillin and streptomycin (HyClone, Thermo Scientific), phosphate-buffered saline (PBS), dimethyl sulfoxide (DMSO), lipopolysaccharide (Sigma, USA), and indomethacin (TMstandard, USA) were purchased from Sino-American Biotechnology Company (Beijing, China); ultrapure water was obtained from Gen Pure (Thermo, USA). 3-(4,5-Dimethylthiazol-2-yl)-2,5-diphenyltetrazolium bromide (MTT) and dimethyl sulfoxide (DMSO) were purchased from Sangon Biotech (Shanghai, China). The Nitric Oxide (NO) Assay kit was purchased from Nanjing Jiancheng Bioengineering Institute (Nanjing, China). *p*-Nitrophenol α-D-glycopyranoside (pNPG) and α-glucosidase (EC 3.2.20, from *Saccharomyces cerevisiae*, lyophilized powder) were obtained from Sigma (USA).

#### Chemicals and Reagents

Acetonitrile (HPLC grade) was purchased from Fisher Scientific (Fisher Scientific, USA). Purified water was obtained using a Milli-Q system (Millipore, USA). All other reagents were of analytical grade. The reference compounds of 18 analytes were purchased from the National Institute for the Control of Pharmaceutical and Biological Products (Beijing, China).

### Methods

#### Extraction and Processing

Dried whole-plant powder of *C. daurica* (1.45 kg) was extracted successively with 70% ethanol to produce 362.5 g of extract. The extract was dissolved in water, and then extracted successively with petroleum ether, ethyl acetate, and n-butanol to produce 55.91, 21.47, and 110.77 g of dry extract, respectively. The aqueous phase contained 174.26 g of the residue.

#### Anti-Inflammatory Assay

##### Cell Culture

RAW 264.7 mouse macrophages were cultured in high glucose DMEM supplemented with 10% FBS and a mixture of penicillin and streptomycin. The cells were maintained in a humidified incubator with an atmosphere of 5% CO_2_ at 37°C.

##### Cell Viability Assay

The MTT assay was used to assess whether *C. daurica* extracts exerted cytoprotective effects on RAW 264.7 cells. RAW 264.7 cells were seeded in a 96-well plate at a density of 1 × 10^5^ cells/mL, cultured for 24 h, and then divided into control and treatment groups. The four extracts of *C. daurica* were dissolved in DMSO (<0.01%) and diluted with complete culture medium. The treatment cell groups were pretreated with vehicle alone or with different concentrations (25, 50, 100, 200, 400, 800, 1,600, and 3,200 μg/mL) of *C. daurica* extracts (water, n-butanol, ethyl acetate, and petroleum-ether extracts) for 24 h. The cells were then incubated with 10 µL of MTT solution for 4 h at 37°C, after which the supernatant was discarded and 150 µL of DMSO was added to each well. The plates were oscillated at a low speed for 5 min at room temperature until all the formed crystals were fully dissolved. Optical density was determined at an absorbance wavelength of 570 nm (Thermo Scientific Multiskan FC, Thermo, USA). The optical density of the control (untreated) cells was designated as 100% viability.

##### Nitric Oxide Assay

RAW 264.7 cells were plated at a density of 5 × 10^5^ cells/mL in 24-well plates, cultured for 12 h, and then treated with LPS (1 μg/mL) and incubated for 4 h. Thereafter, the cells were treated with different concentrations (25, 50, 100, 200, and 400 μg/mL) of *C. daurica* extracts (water, n-butanol, ethyl acetate, and petroleum-ether extracts) and incubated for an additional 20 h. Meanwhile, the negative control groups were treated for 20 h with 400 μg/mL of each extract, but not with LPS. Indomethacin (6.25, 12.5, 25, 50, and 100 μM) was used as the positive control (Cheng et al., 2017). The amount of NO in the medium was detected using the Griess test. One hundred microliters of each supernatant were mixed with equal volume of Griess reagent (1% sulfanilamide in 5% phosphoric acid and 0.1% naphthylethylenediamine dihydrochloride in water). The absorbance of the mixture at 550 nm and concentration of nitrite were determined using serially diluted sodium nitrite as the standard (Qiao et al., 2005).

#### α-Glucosidase Inhibition Assay

We examined whether the four *C. daurica* extracts could inhibit the activity of α-glucosidase using a previously published method (Schmidt et al., 2014). Briefly, each extract of *C. daurica* was dissolved in DMSO (< 0.05%), and then diluted with phosphate buffer (pH 6.9). Then, 20 µL of each extract (at a concentration of 50, 75, 100, 125, or 150 μg/mL) was added into a 96-well microplate. Next, 10 µL of α-glucosidase from *Saccharomyces*, 10 µL of 3 µM glutathione, and 20 μL of phosphate buffer were added to each well, and the plate was incubated at 37°C for 10 min. Thereafter, 20 μL of 0.01 M p-nitrophenyl glucopyranoside (pNPG) was added to each well to quench the reaction, and the plate was incubated at 37°C for 20 min. The reaction was stopped by the addition of 100 µL of 0.1 M Na_2_CO_3_ into each well, and the absorbance was recorded at 405 nm. The control sample contained buffer instead of α-glucosidase. Acarbose was used as the positive control.

#### Isolation of Pure Compounds

The n-butanol extract (25 g) was fractionated on a D101 macroporous resin column using EtOH–H_2_O (70:30) to obtain 15 fractions, and then by TLC to obtain four main fractions (Frs. A1–A4). Fr. A1 was recrystallized repeatedly (MeOH–EtOH) to obtain compound 1 (57.9 mg). Fr. A2 was further purified *via* repeated chromatography on Sephadex LH-20 to obtain compound 2 (11.3 mg). Fr. A3 was further purified by chromatography on an ODS column (MeOH–H_2_O, 0:100–20:80) to obtain four fractions (Frs. B1–B4). Fr. B1 was further purified by repeated chromatography on silica gel to obtain compound 3 (25.4 mg). Fr. B2 was further purified by chromatography on an MCI column (MeOH–H_2_O, gradient elution) to obtain compound 4 (14.0 mg). Fr. B3 was further purified by repeated chromatography on Sephadex LH-20 to obtain compound 5 (16.6 mg). Fr. B4 was further purified by repeated chromatography on Sephadex LH-20 to obtain compound 6 (27.0 mg). Fr. A4 was further purified by chromatography on an ODS column (MeOH–H_2_O (30:70–70:30) to obtain seven fractions (Frs. C1–C7). Combined Fr. C4 was further purified by repeated chromatography on Sephadex LH-20 (MeOH–H_2_O, 70:30) to obtain compounds 7 (119.9 mg) and 8 (19.8 mg).

Ethyl acetate extract (17 g) was fractionated on a D101 macroporous resin column using EtOH–H_2_O (70:30) to obtain 10 main fractions (Frs. D1–D10) [10]. Frs. D4–D7 was further purified by chromatography on an ODS column (MeOH–H_2_O, 30:70–70:30) to obtain seven fractions (Frs. E1–E7). Combined Fr. E4 was further purified by repeated chromatography on Sephadex LH-20 (MeOH–H_2_O, 70:30) to obtain compounds 7 (74.0 mg) and 8 (22.0 mg). Frs. D8–D9 was further purified by chromatography on silica gel (CHCl_3_–MeOH, 90:10–60:40) to obtain 12 fractions (Frs. F1–12). Combined Frs. F2–F6 was further purified by repeated chromatography on Sephadex LH-20 (MeOH–H_2_O, 80:20) to obtain compounds 9 (20.4 mg) and 10 (23.7 mg).

#### HPLC-MS

##### HPLC–MS/MS System and Operating Conditions

The Thermo Scientific™ Ultimate™ 3000 RS system was used in this assay. Chromatographic separation was performed on the Hypersil GOLD C_18_ (100 mm × 2.1 mm, 3 μm; Thermo Scientific) column. Acetonitrile (solvent B) and water (containing 0.1% formic acid, solvent A) were used as the mobile phase. The gradient was as follows: 0–35 min, 5%–60% B. The flow rate was 0.3 mL/min.

Mass spectrometry data were acquired using the Q Exactive High-Resolution Benchtop Quadrupole Orbitrap Mass Spectrometer (Thermo Fisher Scientific, USA) equipped with an ESI interface in the positive and negative ion modes. Optimal parameters were as follows: probe heater temperature, 350°C; spray voltage, 3.5 kV for the positive and negative ion modes; sheath gas, 35 arb; auxiliary gas, 10 arb. Capillary temperature was set at 320°C and S-lens was 50 V. Full-scan MS data were generated across a mass range of 100–1500 Da. The stepped normalized collision energy setting was 20, 40, and 60 eV. All MS spectra were acquired using the Q Exactive Mass Spectrometer controlled using Xcalibur 3.0 software (Thermo Fisher Scientific).

##### Data Processing

MS data were processed using QualBrowser of Xcalibur 3.0 software (Thermo Fisher Scientific), and they included extracted ion chromatograms, fragmentation behavior, and elemental compositions with mass errors within 5 ppm. All analytes were identified using their elemental composition, accurate mass measurement, elution order, fragmentation behavior, fragmentation pattern of the standard compound, and comparison with reliable data in compounds database (Wang et al., 2018).

#### HPLC Analysis

The nucleosil C18 column (4.6 mm × 250 mm, 5 μm; Agilent) was used for the analysis of catalpol, verbascoside, isoacteoside, luteolin, and apigenin by HPLC. The mobile phase consisted of water (containing 0.1% phosphoric acid, solvent A) and acetonitrile (solvent B). The gradient was as follows: 0–15 min, 1% B; 15–25 min, 1–15% B; 25–70 min, 15–35% B; 70–75 min, 35–50% B. The flow rate was 1 mL/min. Injection volume was 10 μL, and the temperature of the column was maintained constant at 30°C. Detection was performed using at wavelengths of 210, 330, and 365 nm. The n-butanol and ethyl acetate extracts of *C. daurica* were analyzed by HPLC. Each sample was assayed in triplicate. Chromatographic peaks of the samples were quantified *via* external standard method. The HPLC method developed in this study was validated for linearity, precision, repeatability, and stability.

#### Statistical Analysis

All data were analyzed using SPSS 19.0, and the results are presented as mean ± standard deviation (SD) of three independent experiments. Values with *p* < 0.05 were considered significantly different.

## Results

### Anti-Inflammatory Assay

#### Effect of the Four *C. daurica* Extracts on the Viability of RAW 264.7 Cells

**Figure 2:**
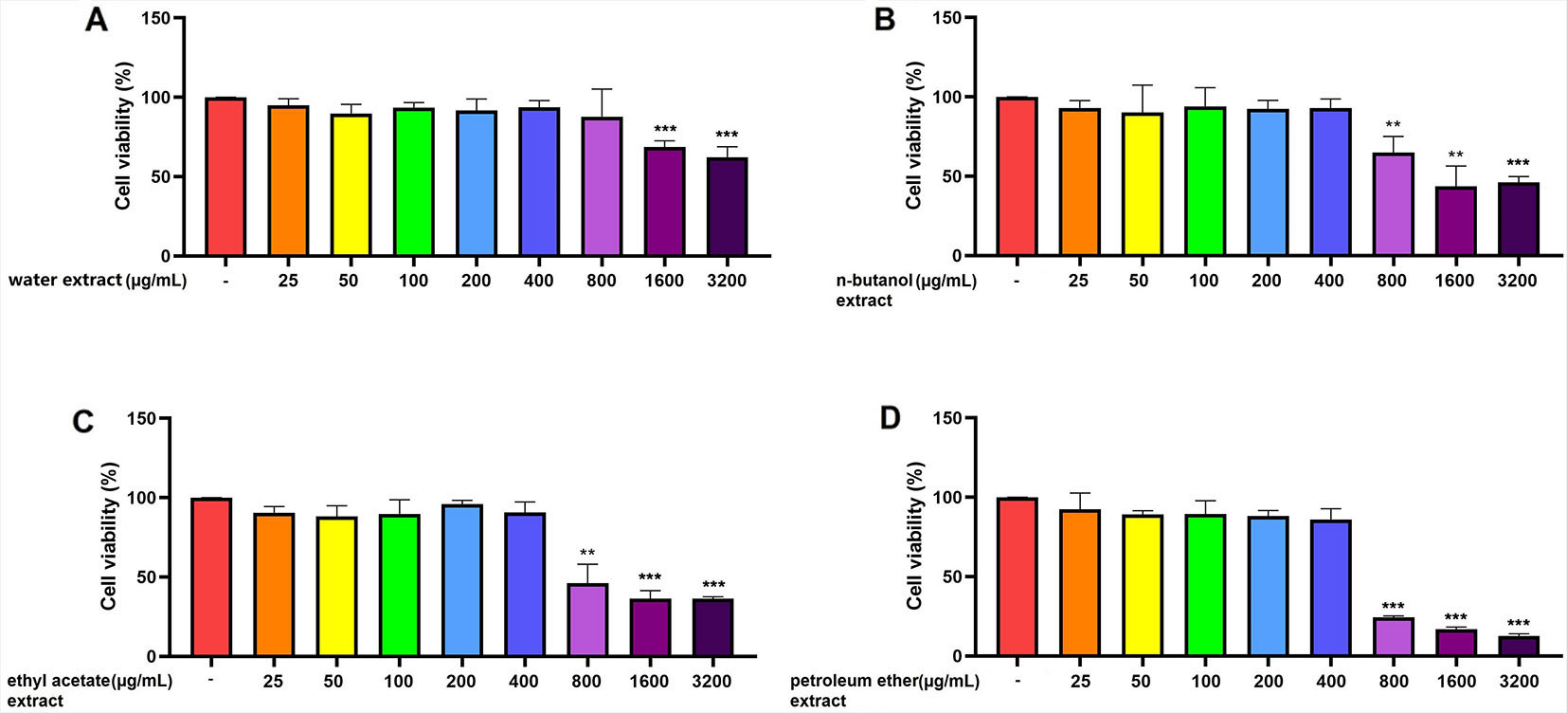
Effect of the four *C. daurica* extracts on viability of RAW 264.7 cells. **(A)** water extract. **(B)** n-butanol extract. **(C)** ethyl acetate extract. **(D)** petroleum ether extract. Data are presented as means ± SD, *n* = 5. **p < 0.01, ***p < 0.001 compared with control.

RAW 264.7 cells, pretreated for 24 h with five different concentrations (25, 50, 100, 200, and 400 μg/mL) of the water, n-butanol, ethyl acetate, and petroleum-ether extracts showed no significant differences in cell viability compared with that of the control group (*p* > 0.05) (**Figure 2**). However, pretreatment with 800, 1,600, or 3,200 μg/mL of the water, n-butanol, ethyl acetate, or petroleum-ether extract significantly decreased cell activity (*p* < 0.01). These results indicate that pretreatment of RAW 264.7 cells with the four *C. daurica* extracts at concentrations of 25, 50, 100, 200, and 400 μg/mL did not result in cytotoxicity; therefore, these concentrations were selected for further experiments.

#### Effect of Extracts From Four Parts of *C. daurica* on RAW 264.7 Cell NO Production

**Figure 3:**
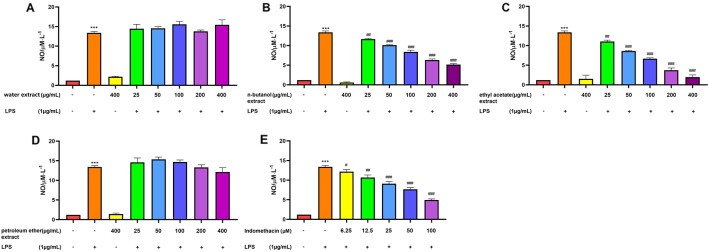
Effect of extracts from four parts of the *C. daurica* on LPS-induced NO production in RAW 264.7 cell. **(A)** water extract. **(B)** n-butanol extract. **(C)** ethyl acetate extract. **(D)** petroleum ether extract. **(E)** indomethacin. Data are presented as means ± SD, *n* = 5. ***p < 0.001 compared with control; ^###^ *p* < 0.001, ^##^ *p* < 0.01, ^#^ *p* < 0.05 compared with LPS. (Control group; Model group: LPS; Negative control group: water extract, n-butanol extract, ethyl acetate extract, petroleum ether extract; Treated group: water extract + LPS, n-butanol extract + LPS, ethyl acetate extract + LPS, petroleum ether extract + LPS).

As shown in **Figure 3**, RAW 264.7 cells did not release NO in response to the culture medium. In this study, LPS was used to activate RAW 264.7 cells. NO production by RAW 264.7 cells stimulated with LPS (1 μg/mL) was significantly increased compared with that of the control group (*p* > 0.001). When used separately, the four extracts of *C. daurica* (400 μg/mL) did not affect NO production in RAW 264.7 cells (*p* > 0.05). However, when different concentrations of n-butanol and ethyl acetate extracts (25, 50, 100, 200, and 400 μg/mL) were added to the culture media during cell stimulation (20 h), NO production was inhibited in a dose-dependent manner (*p* < 0.05, **Figures 3B, C**). The n-butanol and ethyl acetate extracts exhibited significant anti-inflammatory activity with the IC_50_ values of 197.00 ± 0.74 and 90.00 ± 1.05 μg/mL, respectively, whereas, indomethacin, as the positive control, had an IC_50_ of 59.97 ± 0.80 µM (**Table 1**). However, the water and petroleum-ether extracts did not inhibit LPS-induced NO production and did not exhibit anti-inflammatory activity (**Figures 3A, D**). These results suggest that the extracts of *C. daurica* may function, at least partially, *via* macrophage activation in the host defense response.

**Table 1:** IC_50_ values (µg/mL) of inhibitory activities of *C. daurica* extracts on LPS-induced NO production in RAW264.7 cell. (n = 5,x ± s).

Source	Extract fractions	IC_50_
*C. daurica*	Water extract	–
	n-butanol extract	197.00 ± 0.74
	Ethyl acetate extract	90.00 ± 1.05
	Petroleum ether extract	–
Indomethacin^a^	–	59.97 ± 0.80^b^

a Indomethacin was used as positive control.

b The concentration units were μM.

### α-Glucosidase Inhibition

To the best of our knowledge, this is the first study to report that the extracts of *C. daurica* can inhibit the activity of α-glucosidase. The results of the effects of *C. daurica* extracts on α-glucosidase activity are presented in **Table 2**. As shown in **Table 2**, the water, n-butanol, ethyl acetate, and petroleum-ether extracts of *C. daurica* exerted inhibitory activity on the activity of α-glucosidase (IC_50_ = 175.8 ± 2.11, 133.2 ± 2.59, 105.9 ± 0.89, and 173.6 ± 1.11 µg/mL, respectively). Among the four extracts of *C. daurica*, ethyl acetate extract exerted significant inhibitory effects on α-glucosidase, showing an IC_50_ of 105.9 ± 0.89 μg/mL; the α-glucosidase-inhibition potency was equivalent to that of acarbose (108.2 ± 1.28 μg/mL, *p* > 0.05). These results show that *C. daurica* extracts can be used to design new plant-based drugs and nutraceuticals.

**Table 2:** IC_50_ values (µg/mL) of α-glucosidase inhibition activity of *C. daurica* extracts. (n = 3,x ± s).

Source	Extract fractions	IC_50_
*C. daurica*	Water extract	175.8 ± 2.11
	n-butanol extract	133.2 ± 2.59
	Ethyl acetate extract	105.9 ± 0.89
	Petroleum ether extract	173.6 ± 1.11
Acarbose	–	108.2 ± 1.28

### Isolation of Pure Compounds

**Figure 4:**
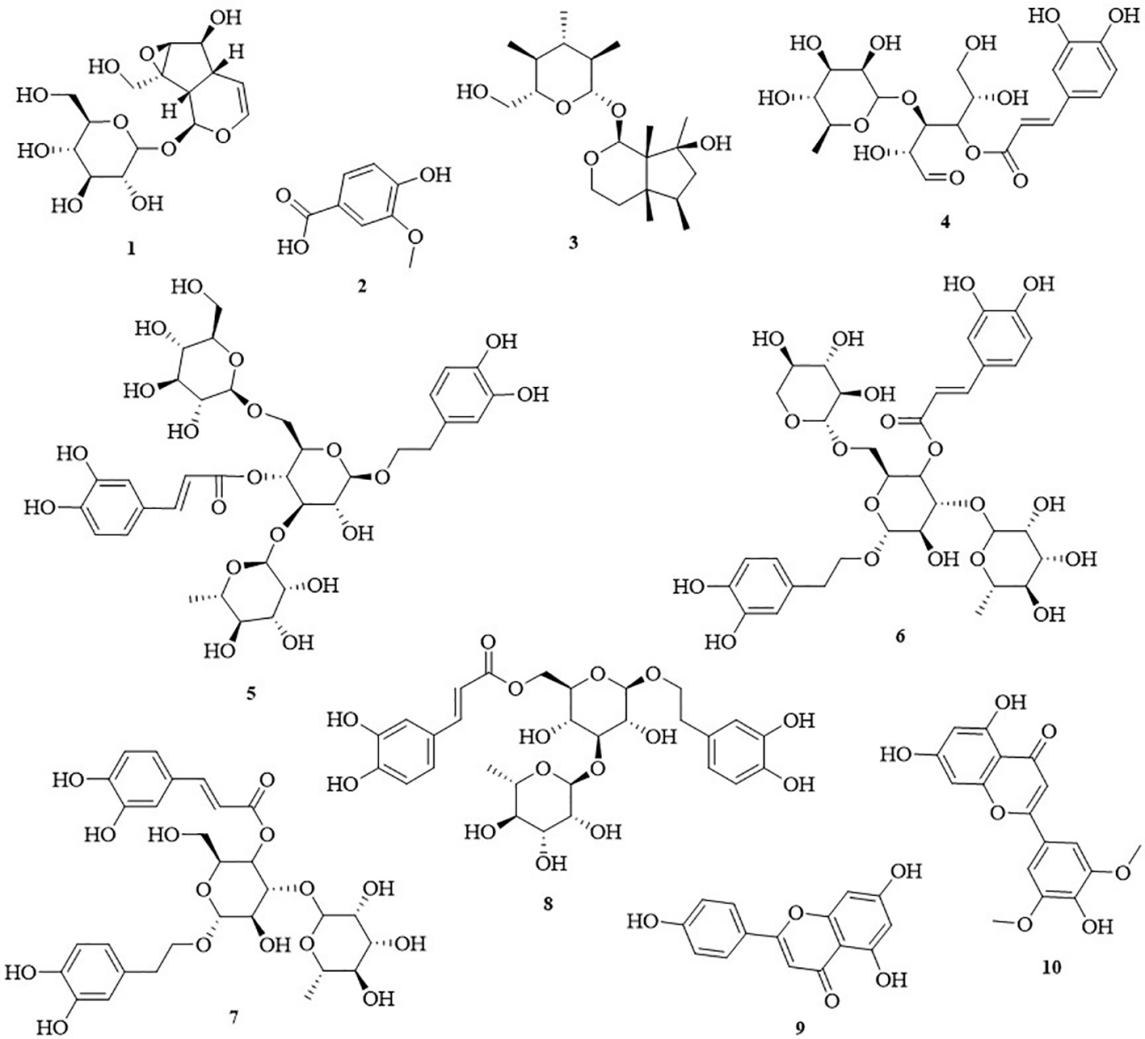
The chemical structures of ten monomeric compounds. 1 catalpol; 2 vanillic acid; 3 ajugol; 4 cistanoside F; 5 echinacoside; 6 arenarioside; 7 verbascoside; 8 isoacteoside; 9 apigenin; 10 tricin.

The n-butanol and ethyl acetate extracts showed considerable anti-inflammatory and α-glucosidase-inhibitory activities. Furthermore, we conducted phytochemical analysis of the n-butanol and ethyl acetate extracts from *C. daurica*. We isolated compounds 1, 2, 3, 4, 5, 6, 7, and 8 from the n-butanol extract. Compounds 7, 8, 9, and 10 were isolated from the ethyl acetate extract. The structure of these compounds was identified by comparing their spectral data (obtained *via* proton nuclear magnetic resonance [^1^H-NMR, ^13^C-NMR] (the spectral data of compounds 1–10 are shown in **Supplemental Material**), and electrospray ionization [ESI-MS]) with published data on catalpol (1) (Nguyen et al., 2005), vanillic acid (2) (Chen et al., 2011), ajugol (3) (Zhang et al., 2016a), cistanoside F (4) (Liu et al., 2016), echinacoside (5) (Zhen and Shi, 2004), arenarioside (6) (Andary et al., 1985), verbascoside (7) (Wu et al., 2012), isoacteoside (8) (Liu et al., 2011), apigenin (9) (Chu et al., 2014), and tricin (10) (Han et al., 2013). The chemical structure of the 10 compounds is shown in **Figure 4**. Our study is the first to isolate compounds 2, 4, 5, 6, 7, 8, and 10 from *C. daurica*. Furthermore, this is the also first report of phenylethanoloside compounds found in *C. daurica*.

### HPLC-MS Analysis

**Figure 5:**
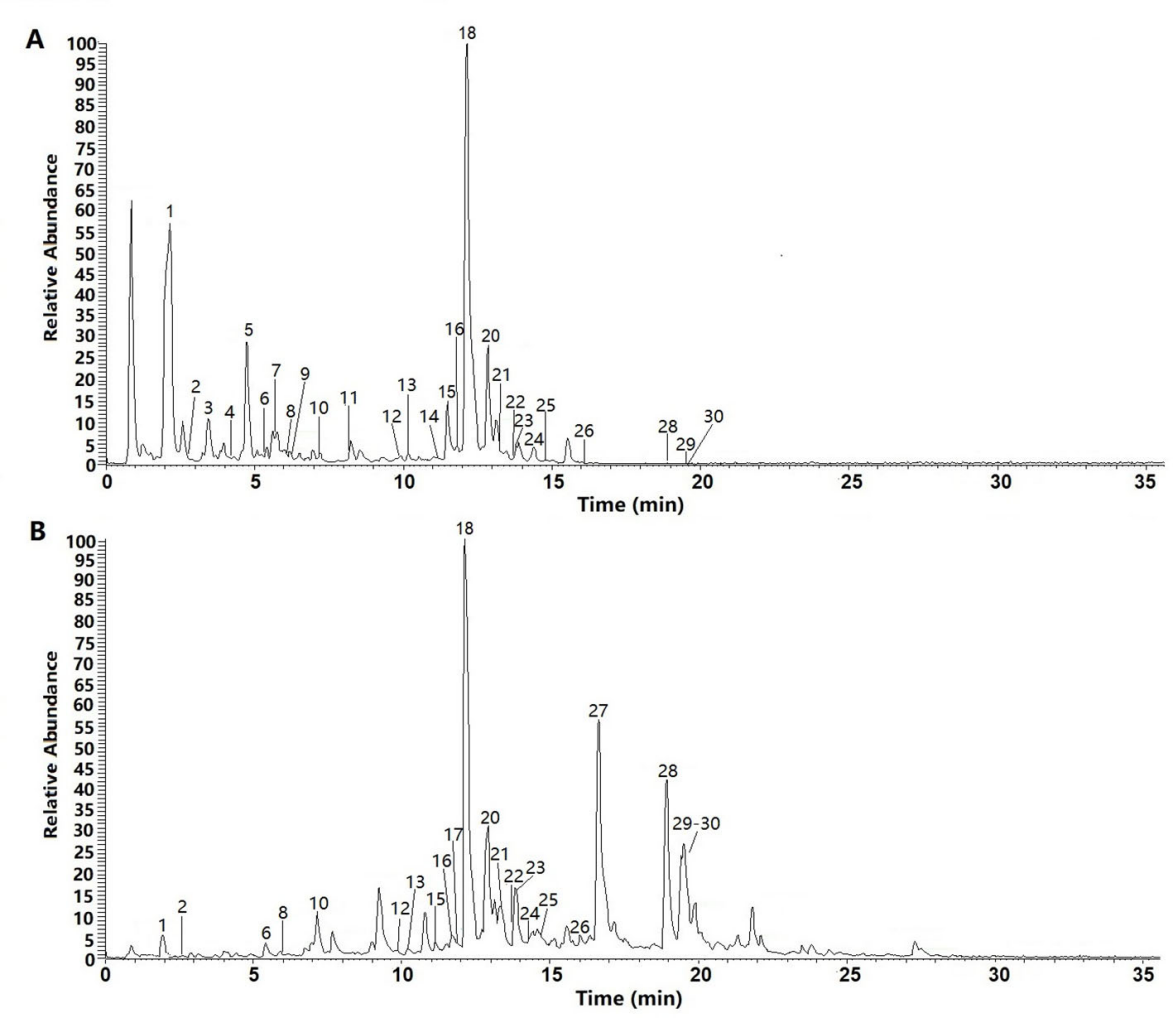
The total ion chromatograms (TIC) of n-butanol extract **(A)** and ethyl acetate extract **(B)** form the *C. daurica* in negative mode.

We examined the chemical composition of *C. daurica*. Chemical composition of the n-butanol and ethyl acetate extracts of *C. daurica* was determined by UHPLC-Q-Exactive. To determine the chemical composition, we evaluated the retention time, ionic species, and major fragments in MS and MS^2^ spectra. We also searched an online database and literature, and examined fragmentation patterns of standard compounds. Based on these analyses, the chemical structure of the n-butanol and ethyl acetate extracts of *C. daurica* was tentatively identified and classified into four major groups, namely, phenylethanoid glycosides, flavonoids, iridoids, and phenolic acids. The total ion chromatograms of n-butanol and ethyl acetate extracts of *C. daurica*, both in the negative ion mode, are presented in **Figure 5**. Retention time, molecular formula, theoretical mass, and measured mass of the 30 identified compounds are presented in **Table 3**.

**Table 3:** Identification of major chemical constituents in n-butanol extract and ethyl acetate extract form the *C. daurica*.

Peak	Compounds	Formula	Ion mode	Theoretical (*m/z*)	n-butanol extract		Ethyl acetate extract	
					**RT (min)**	Measured (*m/z*)	**RT (min)**	Measured (*m/z*)
1	Catalpol*	C_15_H_22_O_10_	Negative	361.11402	2.15	361.11423	2.00	361.11420
2	Isoxepac glucuronide	C_22_H_20_O_10_	Negative	443.09837	2.58	443.09638	2.56	443.24743
3	Aucubin*	C_15_H_22_O_9_	Negative	345.11910	3.48	345.11911	–	–
4	Vanillic acid*	C_8_H_8_O_4_	Negative	167.03498	4.29	167.03422	–	–
5	Ajugol*	C_15_H_24_O_9_	Negative	347.13475	4.72	347.13461	–	–
6	Loganic acid*	C_16_H_24_O_10_	Negative	375.12967	5.37	375.12974	5.37	375.12992
7	1β-methoxylgardendiol	C_10_H_14_O_5_	Negative	213.07684	5.73	213.07636	–	–
8	Cistanoside F*	C_21_H_28_O_13_	Negative	487.14571	6.02	487.30565	5.94	487.07273
9	Gentiopicroside	C_16_H_20_O_9_	Negative	355.10345	6.39	355.10373	–	–
10	Caffeic acid*	C_9_H_8_O_4_	Negative	179.03498	7.19	179.03583	7.13	179.03406
11	1β-hydroxyl-4-epigardendiol	C_11_H_16_O_5_	Negative	227.09249	8.24	227.07684	–	–
12	Campneoside II	C_29_H_36_O_16_	Negative	639.19305	9.89	639.19274	9.88	639.19316
13	Echinacoside*	C_35_H_46_O_20_	Negative	785.25096	10.16	785.25056	10.17	785.25104
14	Rutin*	C_27_H_30_O_16_	Negative	609.14610	11.20	609.14640	–	–
15	Arenarioside*	C_34_H_44_O_19_	Negative	755.24040	11.49	755.23968	11.48	755.24013
16	Cynaroside*	C_21_H_20_O_11_	Negative	447.09328	11.75	447.09342	11.76	447.12989
17	Rehmapicroside	C_16_H_26_O_8_	Negative	345.15549	–	–	11.84	345.15567
18	Verbascoside*	C_29_H_36_O_15_	Negative	623.19814	12.14	623.19793	12.12	623.19875
19	4-Coumaric acid*	C_9_H_8_O_3_	Negative	165.05462	12.37	165.05488	12.35	165.11338
20	Isoacteoside*	C_29_H_36_O_15_	Negative	623.19814	12.83	623.19793	12.91	623.19875
21	Apigenin-7-*O*-*β*-D-glucuronide	C_21_H_20_O_10_	Negative	431.09837	13.3	431.19252	13.27	431.09855
22	Tricin-7-*O*-*β*-D-glucuronide	C_23_H_24_O_12_	Negative	491.11949	13.76	491.15596	13.78	491.19275
23	Cistanoside C	C_30_H_38_O_15_	Negative	637.21379	13.83	637.10461	13.85	637.21399
24	Chrysoeriol-7-*O*-*β*-D–glucuronide	C_22_H_22_O_11_	Negative	461.10893	14.40	461.16673	14.32	461.14572
25	Jionoside C	C_29_H_36_O_13_	Negative	591.20831	14.69	591.07978	14.68	591.26153
26	Martynoside	C_31_H_40_O_15_	Negative	651.22944	16.02	651.19323	16.03	651.19342
27	Luteolin*	C_15_H_10_O_6_	Negative	285.04046	–	–	16.64	285.04056
28	Apigenin*	C_15_H_10_O_5_	Negative	269.04554	18.94	269.04461	18.93	269.04565
29	Tricin*	C_17_H_14_O_7_	Negative	329.06667	19.45	329.08803	19.44	329.23330
30	Chrysoeriol*	C_16_H_12_O_6_	Negative	299.05611	19.55	299.07734	19.54	299.05626

* Compared with standard compounds.

Individual compounds in the n-butanol and ethyl acetate extracts of *C. daurica* were characterized as follows. Peak 1 in the n-butanol and ethyl acetate extracts was observed at *m/z* 361. According to Song et al. (2016), peak 1 was characterized as catalpol, consistent with the fragmentation pattern of the standard compound. Based on MS, MS/MS data, data found in the literature (Song et al., 2016), and comparison with fragmentation patterns of the standard compounds, vanillic acid (peak 4) was detected in the n-butanol extract of *C. daurica*. Vanillic acid showed fragments at *m/z* 167, 151, and 107. Peak 18, at *m/z* 623, was identified as verbascoside because it showed fragments at *m/z* 461 and 161. This fragmentation pattern was consistent with that of the standard compound, as well as patterns reported in previous studies (Song et al., 2016; Zhang et al., 2016b). Peak 20 also showed similar fragmentation pattern and was proposed to be a verbascoside isomer. Based on comparison with fragmentation pattern of the standard compound, Peak 20 was identified as isoacteoside. Peak 29, with *m/z* 329, was proposed to be tricin and showed fragments of *m/z* 314, 162, and 151 in the MS/MS spectrum. These MS^2^ data matched the fragmentation pattern of the standard compound.

Aucubin, caffeic acid, rutin, cynaroside, catalpol echinacoside, and apigenin, previously reported as present in *C. daurica*, show strong anti-inflammatory and hypoglycemic activities (Sun et al., 2004; Li, 2013; Dong and Chen, 2013; Han et al., 2017; Li et al., 2017; Yang et al., 2017; Bao et al., 2018), whereas vanillic acid, luteolin, verbascoside, and tricin show considerable anti-inflammatory activity (Jeong et al., 2017; Yin et al., 2017; Li et al., 2018a; Li et al., 2018b).

### HPLC Analysis

**Figure 6:**
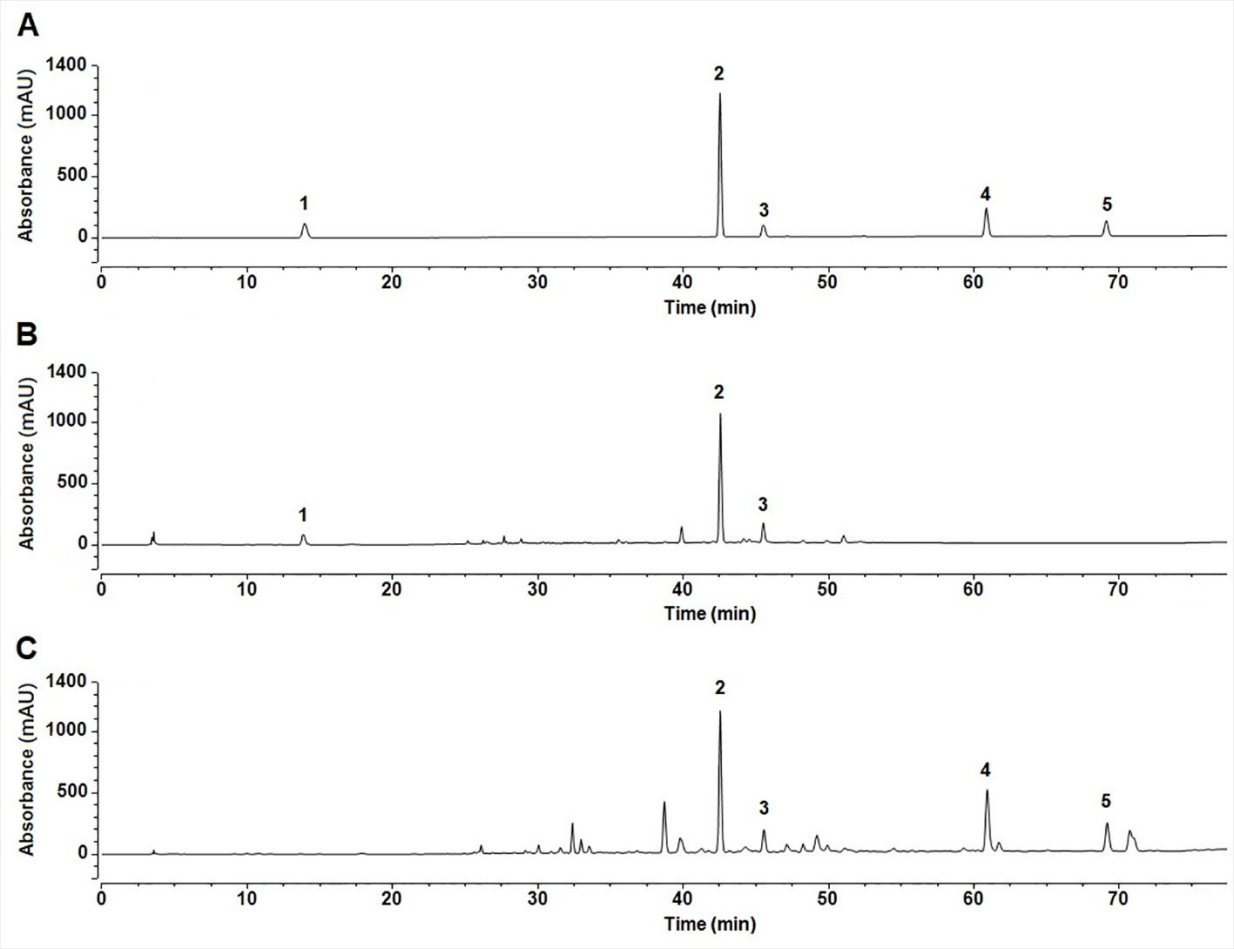
The fingerprints of the five compounds in different extracts of *C. daurica*. **(A)** Mixed standard. **(B)** n-butanol extract. **(C)** ethyl acetate extract. 1 catalpol; 2 verbascoside; 3 isoacteoside; 4 luteolin; 5 apigenin.

Based on the biological activity and chemical composition of the n-butanol and ethyl acetate extracts, identified by HPLC-MS, we developed a novel HPLC method for simultaneous identification of five compounds with high content and potential activity. A sample chromatogram is shown in **Figure 6**. As illustrated in **Figure 6**, the five compounds were well separated under the described chromatographic conditions. As shown in **Table 4**, all calibration curves exhibited excellent linearity (R^2^ = 0.9991–0.9998) in a relatively wide concentration range. This method was validated for precision, repeatability, and stability, as shown in **Table 5**. These results show that the HPLC method, developed in this study, was precise, accurate, and sensitive enough for simultaneous quantitative evaluation of five compounds in *C. daurica*. This novel HPLC method was subsequently used for simultaneous identification of five compounds in the n-butanol and ethyl acetate extracts of *C. daurica*. As shown in **Table 6**, the content of each compound varied depending on the extract; for example, the content of verbascoside in the n-butanol extract and that in ethyl acetate extract was 114.263 ± 0.255 and 162.955 ± 0.203 mg/g, respectively.

**Table 4:** Calibration curve for five compounds.

Compound	Calibration curve^a^	R^2^	Linear range (µg/mL)
Catalpol	y = 45.201x + 0.6434	0.9997	0.472–2.832
Verbascoside	y = 264.5x + 28.613	0.9991	0.346–2.076
Isoacteoside	y = 636.89x + 0.5168	0.9998	0.022–0.132
Luteolin	y = 1123.9x + 0.538	0.9995	0.028–0.168
Apigenin	y = 1083.6x + 0.2633	0.9995	0.018–0.108

a y = peak area and x = concentration (µg/mL).

**Table 5:** Precision, repeatability, and stability of five compounds (RSD%, n = 5).

Compound	Precision RSD	Repeatability RSD (n-butanol extract)	Repeatability RSD (Ethyl acetate extract)	Stability RSD (n-butanol extract)	Stability RSD (Ethyl acetate extract)
Catalpol	0.09	0.31	–	1.71	–
Verbascoside	0.89	0.33	1.32	0.37	0.84
Isoacteoside	0.15	0.53	1.35	0.61	0.48
Luteolin	0.59	–	0.97	–	1.02
Apigenin	0.43	–	1.93	–	1.12

**Table 6:** Quantitative analytical results of n-butanol extract and ethyl acetate extract form the *C. daurica* (mg/g, n = 3,x ± s).

Extract fractions	Catalpol	Verbascoside	Isoacteoside	Luteolin	Apigenin
n-butanol extract	113.918 ± 0.127	114.263 ± 0.255	9.475 ± 0.059	–	–
Ethyl acetate extract	–	162.955 ± 0.203	15.967 ± 0.021	27.701 ± 0.026	14.079 ± 0.004

## Discussion

Recently, Mongolian medicines have been more commonly used, owing to their low adverse effect; they also protect against and treat certain diseases. Furthermore, research on approaches that help explore the therapeutic value of natural pharmacotherapeutic agents has become a growing trend (Chester et al., 2017). *C. daurica* is widely used in traditional Mongolian medicine for the treatment of impetigo, psoriasis, pruritus, fetotoxicity, and diabetes. In spite of its tremendous therapeutic potential, complete chemical signature of *C. daurica* is unknown. Therefore, the aim of this study was to assess bioactivity and analyze chemical composition of *C. daurica* to lay a foundation to identify new drugs.

Based on the traditional therapeutic effect of *C. daurica*, the anti-inflammatory and α-glucosidase-inhibitory activities of four polar *C. daurica* extracts were preliminarily evaluated to identify the active extracts. None of the tested samples exhibited cytotoxic effects at the effective concentrations (**Figure 2**). The n-butanol and ethyl acetate extracts showed pronounced inhibitory effects on LPS-induced NO production in RAW 264.7 cells compared with that of indomethacin (**Table 1**). Furthermore, the four extracts of *C. daurica* (400 μg/mL) used separately did not affect NO production in RAW 264.7 cells (*p* > 0.05) (**Figure 3**). Activated macrophages release inflammatory mediators such as NO. The excessive production of inflammatory mediators in prolonged inflammation can cause cellular and tissue damage (Novilla et al., 2017). NO overproduction leads to cellular responses including apoptosis and necrosis (Nagai et al., 2003). To prevent the adverse effect of prolonged inflammation, anti-inflammatory agents are needed. Any substance that inhibits the production of these pro-inflammatory molecules is considered a potential anti-inflammatory agent (Dinarello, 2010). Thus, the active extracts of *C. daurica* were n-butanol and ethyl acetate extracts.

In this study, the ethyl acetate extract significantly inhibited α-glucosidase (with IC_50_ of 105.9 ± 0.89 μg/mL), and the α-glucosidase-inhibition potency was equivalent to that of acarbose (108.2 ± 1.28 μg/mL) (**Table 2**). n-Butanol presented the second highest inhibitory activity, with the IC_50_ value of 133.2 ± 2.59 μg/mL. The therapeutic action of *C. daurica* extracts can be attributed to their inhibition activity against α-glucosidase, thus reducing postprandial hyperglycemia and controlling diabetes. Acarbose is an inhibitor of carbohydrate digesting enzyme used to decrease glucose absorbance, but it has some gastrointestinal adverse effects (Di Carli et al., 2003). Mongolian medicines can inhibit carbohydrate-metabolizing enzymes; due to their almost no adverse effect, studies have focused on *C. daurica* extracts as new α-glucosidase natural inhibitors.

The separation and identification of n-butanol and ethyl acetate extracts from *C. daurica* yielded 10 compounds, namely, catalpol, vanillic acid, ajugol, cistanoside F, echinacoside, arenarioside, verbascoside, isoacteoside, apigenin, and tricin (**Figure 4**). Among them, vanillic acid, cistanoside F, echinacoside, arenarioside, verbascoside, isoacteoside, and tricin were isolated in *C. daurica* for the first time. Previous phytochemical reports described that the main chemical constituents in *C. daurica* are flavonoids and iridoids (Li et al., 2014; Bai et al., 2018). The present study demonstrated for the first time that *C. daurica* contains phenylethanoid glycosides. Based on these results, 30 compounds in n-butanol and ethyl acetate extracts of *C. daurica* were determined by UHPLC-Q-Exactive (**Figure 5** and **Table 3**). The results further proved that the main chemical constituents in *C. daurica* included phenylethanoid glycosides, flavonoids, and iridoids. In addition, this novel HPLC method was subsequently used for the simultaneous identification of five compounds (catalpol, verbascoside, isoacteoside, luteolin, and apigenin) with high content and potential activity in the n-butanol and ethyl acetate extracts of *C. daurica* (**Figure 6**, **Table 4**, and **Table 5**). It is worth noting that the content of verbascoside in the n-butanol and ethyl acetate extracts of *C. daurica* was very high, that is, 114.263 ± 0.255 and 162.955 ± 0.203 mg/g, respectively (**Table 6**). This study provided a chemical basis for the further development and utilization of *C. daurica*.

The anti-inflammatory and anti-diabetic activities of *C. daurica* can be attributed to the bioactive compounds. Phenylethanoid glycosides, flavonoids, and iridoids exhibit extensive pharmacological activities, including anti-inflammatory (Hanfer et al., 2017; Spagnuolo et al., 2018; Zhang et al., 2018) and anti-diabetic activities (Miura et al., 1996; Semaan et al., 2017). Verbascoside has been the subject of biological studies demonstrating anti-inflammatory activity in several models, such as the intestinal inflammation model, where it decreased the presence of pro-inflammatory molecules (Carrillo-Ocampo et al., 2013). Previous studies have shown that verbascoside downregulates some pro-inflammatory signal transduction pathways by increasing the activity of tyrosine phosphatase SHP-1 in U937 cell line (Pesce et al., 2015). Furthermore, studies have reported that intravenous injection of catalpol exhibited anti-hyperglycemic activity in a dose-dependent manner in streptozotocin-induced diabetic rats representing insulin-dependent diabetes mellitus (Huang et al., 2010). Therefore, the development of anti-inflammatory and anti-diabetic drugs from *C. daurica* has considerable advantages. However, understanding the mechanisms underlying the anti-inflammatory and anti-diabetic activities of *C. daurica* needs more studies *in vitro* and *in vivo*. Further studies are also needed to identify the anti-inflammatory and anti-diabetic compounds, and to deeply comprehend the mechanism of action of the active compounds in n-butanol and ethyl acetate extracts of *C. daurica*.

In conclusion, the results of our study showed that phenylethanol-glycoside compounds are important components of *C. daurica*. These findings support the anti-inflammatory and anti-diabetic activities of *C. daurica* and indicate that *C. daurica* could be utilized as a potential source of novel anti-diabetic and anti-inflammatory agents.

## Data Availability Statement

The raw data supporting the conclusions of this article will be made available by the authors, without undue reservation, to any qualified researcher.

## Author Contributions

ML and CZ: Conceived and designed the experiment, analysis and interpretation of data, and critical evaluation of manuscript. XG: literature review, experimental studies, data collection, and manuscript preparation. JW, MZ, PW, CW, RS, EZ, and MZ: literature review, experimental studies. All authors read and approved the final version of the manuscript.

## Funding

This work was supported by the National Natural Science Foundation of China (grant no. 81760776), the National Natural Science Foundation of China (grant no. 81874336), the Natural Science Foundation of Inner Mongolia Autonomous Region (grant no. 2018ZD13), the China Agriculture Research System (grant no. CARS-21), the 2018 Chinese medicine public health service subsidy special “the fourth survey on Chinese materia medica resource” (grant no. Finance Society [2018] 43).

## Conflict of Interest

The authors declare that the research was conducted in the absence of any commercial or financial relationships that could be construed as a potential conflict of interest.
