# Supplementary material for: Bioactivity, Compounds Isolated, Chemical Qualitative, and Quantitative Analysis of Cymbaria daurica Extracts
Source: Front Pharmacol. 2020 Feb 7;11:48. doi: 10.3389/fphar.2020.00048 (PMC7019114; doi:10.3389/fphar.2020.00048)
Supplement: Supplementary file 1 [file DataSheet_1.docx]

Supplementary Material

# Supplementary Data

Compound **1**: catalpol

The spectral data of the compound 1 are shown in **Table 1**.

**TABLE** **1** The ^1^H (600 MHz) and ^13^C-NMR (150 MHz) data of compound 1 in DMSO-*d6*.

| No. | ***δ*_H_ (*J* in Hz)** | ***δ*c** |
| --- | --- | --- |
| 1 | 4.90 (1H, d, *J* = 10.0 Hz) | 93.7 |
| 3 | 6.37 (1H, dd, *J* = 6.0, 2.0 Hz) | 140.7 |
| 4 | 5.01 (1H, dd, *J* = 6.0, 4.5 Hz) | 103.8 |
| 5 | 2.12 (1H, m) | 37.9 |
| 6 | 3.78 (1H, d, *J* = 8.5 Hz) | 77.6 |
| 7 | 3.36 (1H, m) | 61.1 |
| 8 | - | 65.3 |
| 9 | 2.31 (1H, dd, *J* = 10.0, 8.0 Hz) | 42.6 |
| 10 | 3.86 (1H, d, *J* = 13.0 Hz)  3.66 (1H, d, *J* = 13.0 Hz) | 54.9 |
| 1’ | 4.58 (1H, d, *J* = 8.0 Hz) | 98.3 |
| 2’ | 3.02 (1H, t, *J* = 8.5 Hz) | 73.9 |
| 3’ | 3.18 (1H, t, *J* = 8.5 Hz) | 76.8 |
| 4’ | 3.01 (1H, t, *J* = 8.5 Hz) | 70.7 |
| 5’ | 3.13 (1H, m) | 77.9 |
| 6’ | 3.69 (1H, d, *J* = 12.0 Hz)  3.40 (1H, dd, *J* = 12.0, 7.0 Hz) | 61.7 |

Compound **2**: vanillic acid

The spectral data of the compound 2 are shown in **Table 2**.

**TABLE** **2** The ^1^H (600 MHz) and ^13^C-NMR (150 MHz) data of compound 2 in DMSO-*d6*.

| No. | ***δ*_H_ (*J* in Hz)** | ***δ*c** |
| --- | --- | --- |
| 1 | - | 129.2 |
| 2 | 7.23 (2H, m) | 113.2 |
| 3 | - | 147.7 |
| 4 | - | 151.5 |
| 5 | 6.86 (1H, d, *J* =8.6 Hz) | 115.5 |
| 6 | 7.41 (2H, m) | 124.0 |
| 7 | - | 167.7 |
| 8 | 3.82 (3H, s, -OCH_3_) | 56.0 |

Compound **3**: ajugol

The spectral data of the compound 3 are shown in **Table 3**.

**TABLE** **3** The ^1^H (600 MHz) and ^13^C-NMR (150 MHz) data of compound 3 in CD_3_OD.

| No. | ***δ*_H_ (*J* in Hz)** | ***δ*c** |
| --- | --- | --- |
| 1 | 5.49 (1H, d, *J* = 2.0 Hz) | 93.8 |
| 3 | 6.17 (1H, dd, *J* = 6.5, 2.0 Hz) | 140.3 |
| 4 | 4.88 (1H, dd, *J* = 6.5, 2.0 Hz) | 106.3 |
| 5 | 2.73 (1H, d, *J* = 7.5 Hz) | 41.3 |
| 6 | 3.90 (1H, d, *J* = 3.0 Hz) | 79.4 |
| 7 | 2.03 (1H, dd, *J* = 14.0, 5.5 Hz)  1.83 (1H, dd, *J* =14.0, 4.5 Hz) | 50.1 |
| 8 | - | 77.7 |
| 9 | 2.53 (1H, d, *J* = 9.5 Hz) | 52.2 |
| 10 | 1.32 (3H, s) | 25.0 |
| 1’ | 4.67 (1H, d, *J* = 8.0 Hz) | 99.1 |
| 2’ | 3.20 (1H, t, *J* = 1.5 Hz) | 74.8 |
| 3’ | 3.28 (1H, t, *J* = 6.0 Hz) | 78.1 |
| 4’ | 3.29 (1H, t, *J* = 3.0) | 71.6 |
| 5’ | 3.30 (1H, dd, *J* = 5.0, 1.5 Hz) | 78.0 |
| 6’ | 3.67 (1H, dd, *J* = 7.5, 5.5 Hz)  3.31 (1H, dd, *J* = 3.5, 1.5 Hz) | 63.1 |

Compound **4**: cistanoside F

The spectral data of the compound 4 are shown in **Table 4**.

**TABLE** **4** The ^1^H (600 MHz) and ^13^C-NMR (150 MHz) data of compound 4 in CD_3_OD.

| No. | ***δ*_H_ (*J* in Hz)** | ***δ*c** |
| --- | --- | --- |
| 1 | - | 127.9 |
| 2 | 7.00 (1H, brs) | 115.5 |
| 3 | - | 148.1 |
| 4 | - | 148.9 |
| 5 | 6.71 (1H, d, *J* = 8.0 Hz) | 116.8 |
| 6 | 6.90 (1H, d, *J* = 8.0 Hz) | 123.5 |
| 7 | 7.53 (1H, d, *J* = 16.0 Hz) | 146.9 |
| 8 | 6.21 (1H, d, *J* = 16.0 Hz) | 114.9 |
| 9 | - | 168.6 |
| Glucose |  |  |
| 1 | 4.58 (1H, d, *J* = 8.0 Hz) | 94.2 |
| 2 | 3.22 (1H, m) | 70.9 |
| 3 | 3.81 (1H, m) | 79.4 |
| 4 | 4.06 (1H, m) | 70.6 |
| 5 | 3.61 (1H, m) | 76.2 |
| 6 | 3.56 (2H, d, *J* = 2.0) | 62.6 |
| Rhamnose |  |  |
| 1 | 5.18 (1H, brs) | 103.2 |
| 2 | 3.33 (1H, m) | 71.0 |
| 3 | 3.46 (1H, m) | 72.3 |
| 4 | 3.40 (1H, m) | 74.0 |
| 5 | 3.53 (1H, m) | 70.6 |
| 6 | 1.07 (3H, d, *J* = 6.0 Hz) | 18.6 |

Compound **5**: echinacoside

The spectral data of the compound 5 are shown in **Table 5**.

**TABLE 5** The ^1^H (600 MHz) and ^13^C-NMR (150 MHz) data of compound 5 in CD_3_OD.

| No. | ***δ*_H_ (*J* in Hz)** | ***δ*c** |
| --- | --- | --- |
| Aglycone |  |  |
| 1 | - | 130.1 |
| 2 | 6.52 (1H, d, *J* = 1.8 Hz) | 120.4 |
| 3 | - | 115.6 |
| 4 | - | 145.7 |
| 5 | 6.41 (1H, d, *J* = 7.9 Hz) | 144.2 |
| 6 | 6.39 (1H, d, *J* = 7.9, 1.8 Hz) | 115.6 |
| 7 | 2.58 (2H, t, *J* = 7.2 Hz) | 35.9 |
| 8 | 2.72 (2H, m) | 71.1 |
| Caffeoy |  |  |
| 1’ | - | 126.4 |
| 2’ | 6.91 (1H, d, *J* = 1.8 Hz) | 115.6 |
| 3’ | - | 146.3 |
| 4’ | - | 149.2 |
| 5’ | 6.65 (1H, d, *J* = 7.9 Hz) | 115.6 |
| 6’ | 6.87 (1H, dd, *J* = 7.9, 1.8 Hz) | 122.4 |
| 7’ | 7.36 (1H, d, *J* = 15.6 Hz) | 146.7 |
| 8’ | 6.10 (1H, d, *J* = 15.6 Hz) | 115.6 |
| 9’ | - | 168.6 |
| Glucose |  |  |
| 1 | 4.04 (1H, d, *J* = 7.8 Hz) | 103.0 |
| 2 | 3.90 (1H, m) | 74.2 |
| 3 | 3.70 (1H, m) | 79.8 |
| 4 | 3.60 (1H, m) | 77.4 |
| 5 | 3.60 (1H, m) | 73.9 |
| 6 | 3.51 (2H, d, *J* = 2.4 Hz) | 68.9 |
| Glucose |  |  |
| 1 | 4.25 (1H, d, *J* = 7.5 Hz) | 104.2 |
| 2 | 4.10 (1H, m) | 75.2 |
| 3 | 3.99 (1H, m) | 74.1 |
| 4 | 4.87 (1H, m) | 69.9 |
| 5 | 4.39 (1H, m) | 77.8 |
| 6 | 3.36 (2H, d, *J* = 2.4 Hz) | 61.8 |
| Rhamnose |  |  |
| 1 | 4.91 (1H, d, *J* = 1.5 Hz) | 102.1 |
| 2 | 3.50 (1H, m) | 70.4 |
| 3 | 3.30 (1H, m) | 69.9 |
| 4 | 3.20 (1H, m) | 71.4 |
| 5 | 3.30 (1H, m) | 70.4 |
| 6 | 0.84 (3H, d, *J* = 6.0 Hz) | 19.0 |

Compound **6**: arenarioside

The spectral data of the compound 6 are shown in **Table 6**.

**TABLE** **6** The ^1^H (600 MHz) and ^13^C-NMR (150 MHz) data of compound 6 in CD_3_OD.

| No. | ***δ*_H_ (*J* in Hz)** | ***δ*c** |
| --- | --- | --- |
| Aglycone |  |  |
| 1 | - | 131.4 |
| 2 | 6.61 (1H, d, *J* = 2.0 Hz) | 117.1 |
| 3 | - | 145.9 |
| 4 | - | 144.5 |
| 5 | 6.73 (1H, d, *J* = 8.0 Hz) | 116.3 |
| 6 | 6.47 (1H, dd, *J* = 2.0, 8.0 Hz) | 121.3 |
| 7 | 3.63 (1H, m)  3.90 (1H, m) | 72.3 |
| 8 | 2.68 (2H, m) | 36.5 |
| Caffeoy |  |  |
| 1’ | - | 127.5 |
| 2’ | 6.96 (1H, d, *J* = 2.0 Hz) | 115.3 |
| 3’ | - | 146.6 |
| 4’ | - | 149.7 |
| 5’ | 6.69 (1H, d, *J* = 8.0 Hz) | 116.5 |
| 6’ | 6.86 (1H, dd, *J* = 2.0, 8.0 Hz) | 123.3 |
| 7’ | 6.19 (1H, d, *J* = 15.8 Hz) | 114.5 |
| 8’ | 7.51 (1H, d, *J* = 15.8 Hz) | 148.2 |
| 9’ | - | 168.4 |
| Glucose |  |  |
| 1 | 4.27 (1H, d, *J* = 7.9 Hz) | 104.0 |
| 2 | 3.31 (1H, d, *J* = 8.2 Hz) | 75.9 |
| 3 | 3.70 (1H, m) | 81.7 |
| 4 | 4.89 (1H, d, *J* = 9.5 Hz) | 70.3 |
| 5 | 3.63 (1H, m) | 74.6 |
| 6 | 3.49 (2H, d, *J* = 2.0 Hz) | 69.2 |
| Rhamnose |  |  |
| 1 | 5.07 (1H, brs) | 102.9 |
| 2 | 3.83(1H, dd, *J* = 1.8, 3.0 Hz) | 72.2 |
| 3 | 3.49 (1H, m) | 71.9 |
| 4 | 3.21 (1H, d, *J* = 9.6 Hz) | 73.6 |
| 5 | 3.49 (1H, m) | 70.3 |
| 6 | 0.98 (3H, d, *J* = 6.2 Hz) | 18.4 |
| Apiose |  |  |
| 1 | 4.14 (1H, d, *J* =7.5) | 105.1 |
| 2 | 3.13(1H, dd, *J* = 7.6, 8.9 Hz) | 74.7 |
| 3 | 3.23 (1H, d, *J* = 8.9 Hz) | 77.4 |
| 4 | 3.37 (1H, m) | 71.0 |
| 5 | 3.06 (1H, d, *J* = 10.9 Hz)  3.74 (1H, m) | 66.7 |

Compound **7**: verbascoside

The spectral data of the compound 7 are shown in **Table 7**.

**TABLE** **7** The ^1^H (600 MHz) and ^13^C-NMR (150 MHz) data of compound 7 in CD_3_OD.

| No. | ***δ*_H_ (*J* in Hz)** | ***δ*c** |
| --- | --- | --- |
| Aglycone |  |  |
| 1 | - | 131.2 |
| 2 | 6.71 (1H, d, *J* = 2.0 Hz) | 115.1 |
| 3 | - | 146.6 |
| 4 | - | 144.5 |
| 5 | 6.68 (1H, d, *J* = 8.0 Hz) | 117.0 |
| 6 | 6.56 (1H, dd, *J* = 2.0, 8.0 Hz) | 121.0 |
| 7 | 2.76 (2H, m) | 36.4 |
| 8 | 3.88 (2H, m) | 72.1 |
| Caffeoy |  |  |
| 1’ | - | 127.5 |
| 2’ | 7. 05 (1H, d, *J* = 2.0 Hz) | 114.5 |
| 3’ | - | 149.7 |
| 4’ | - | 147.8 |
| 5’ | 6. 77 (1H, d, *J* = 8.5 Hz) | 116.4 |
| 6’ | 6. 95 (1H, dd, *J* = 2.0, 8.5 Hz) | 123.0 |
| 7’ | 7. 59 (1H, d, *J* = 15.5 Hz) | 146.0 |
| 8’ | 6. 26 (1H, d, *J* = 15.5 Hz) | 116.1 |
| 9’ | - | 168.2 |
| Glucose |  |  |
| 1 | 4.39 (1H, d, *J* = 8.0 Hz) | 104.1 |
| 2 | 3.80 (1H, m) | 76.1 |
| 3 | 3.68 (1H, m) | 81.5 |
| 4 | 4.57 (1H, m) | 70.2 |
| 5 | 3.89 (1H, m) | 76.0 |
| 6 | 3.51 (2H, d, *J* = 2.0 Hz) | 62.1 |
| Rhamnose |  |  |
| 1 | 5.19 (1H, brs) | 103.0 |
| 2 | 3.60 (1H, m) | 72.0 |
| 3 | 3.40 (1H, m) | 71.8 |
| 4 | 3.20 (1H, m) | 73.6 |
| 5 | 3.40 (1H, m) | 70.5 |
| 6 | 1.09 (3H, d, *J* = 6.5 Hz) | 18.2 |

Compound **8**: isoacteoside

The spectral data of the compound 8 are shown in **Table 8**.

**TABLE** **8** The ^1^H (600 MHz) and ^13^C-NMR (150 MHz) data of compound 8 in DMSO-*d6*.

| No. | ***δ*_H_ (*J* in Hz)** | ***δ*c** |
| --- | --- | --- |
| Aglycone |  |  |
| 1 | - | 129.4 |
| 2 | 6.61 (1H, s) | 116.0 |
| 3 | - | 143.8 |
| 4 | - | 144.8 |
| 5 | 6.60 (1H, s) | 116.5 |
| 6 | 6.46 (1H, d, *J* = 8.0 Hz) | 119.8 |
| 7 | 2.70 (2H, m | 35.4 |
| 8 | 3.78 (2H, m) | 70.6 |
| Caffeoy |  |  |
| 1’ | - | 125.7 |
| 2’ | 7.07 (1H, d, *J* = 8.8 Hz) | 114.1 |
| 3’ | - | 145.2 |
| 4’ | - | 148.7 |
| 5’ | 6.72 (1H, d, *J* = 8.0 Hz), | 115.7 |
| 6’ | 6.97 (1H, d, *J* = 8.8 Hz) | 121.7 |
| 7’ | 7.47 (1H, d, *J* = 15.6 Hz) | 145.8 |
| 8’ | 6.30 (1H, d, *J* = 16.0 Hz) | 115.1 |
| 9’ | - | 166.8 |
| Glucose |  |  |
| 1 | 4.28 (1H, d, *J* = 6.8 Hz) | 102.9 |
| 2 | 3.60 (1H, m) | 73.9 |
| 3 | 3.10 (1H, m) | 81.0 |
| 4 | 3.30 (1H, m) | 68.4 |
| 5 | 3.79 (1H, m) | 74.4 |
| 6 | 4.14 (2H, d, *J* = 2.4 Hz) | 63.7 |
| Rhamnose |  |  |
| 1 | 5.04(1H, brs) | 100.9 |
| 2 | 3.50 (1H, m) | 70.6 |
| 3 | 3.30 (1H, m) | 70.8 |
| 4 | 3.20 (1H, m) | 72.3 |
| 5 | 3.30 (1H, m) | 68.7 |
| 6 | 1.05 (3H, d, *J* = 7.2 Hz) | 18.1 |

Compound **9**: apigenin

The spectral data of the compound 9 are shown in **Table 9**.

**TABLE** **9** The ^1^H (600 MHz) and ^13^C-NMR (150 MHz) data of compound 9 in DMSO-*d6*.

| No. | ***δ*_H_ (*J* in Hz)** | ***δ*c** |
| --- | --- | --- |
| 2 | - | 164.3 |
| 3 | 6.78 (1H, s) | 104.0 |
| 4 | - | 181.6 |
| 5 | - | 161.5 |
| 6 | 6.23 (1H, d, *J* = 2.0 Hz) | 99.2 |
| 7 | - | 164.1 |
| 8 | 6.52 (1H, d, *J* = 2.0 Hz) | 94.2 |
| 2’ | 7.91 (2H, d, *J* = 8.5 Hz) | 128.7 |
| 3’ | 6.92 (2H, *J* = 8.5 Hz) | 116.2 |
| 4’ | - | 161.2 |
| 5’ | 6.92 (2H, *J* = 8.5 Hz) | 116.2 |
| 6’ | 7.91 (2H, d, *J* = 8.5 Hz) | 128.7 |

Compound **10**: tricin

The spectral data of the compound 10 are shown in **Table 10**.

**TABLE** **10** The ^1^H (600 MHz) and ^13^C-NMR (150 MHz) data of compound 10 in DMSO-*d6*.

| No. | ***δ*_H_ (*J* in Hz)** | ***δ*c** |
| --- | --- | --- |
| 2 | - | 164.0 |
| 3 | 6.99 (1H, s) | 103.4 |
| 4 | - | 181.7 |
| 5 | - | 161.4 |
| 6 | 6.21 (1H, d, *J* = 2.0 Hz) | 98.9 |
| 7 | - | 163.9 |
| 8 | 6.57 (1H, d, *J* = 2.0 Hz) | 95.2 |
| 9 | - | 157.2 |
| 1’ | - | 120.2 |
| 2’ | 7.34 (2H, s) | 104.4 |
| 3’ | 3.89 (6H, s, -OCH_3_) | 148.0 |
| 4’ | - | 139.5 |
| 5’ | 3.89 (6H, s, -OCH_3_) | 148.0 |
| 6’ | 7.34 (2H, s) | 104.4 |

# Supplementary Table

**TABLE** **11** Taxonomic listings.

|  | **Plant common name** | **Plant full scientific name  The Plant List** | **Plant full scientific name**  **Flora of China** |
| --- | --- | --- | --- |
| 1 | Xinba | *Cymbaria daurica* L. | *Cymbaria daurica* L. |
